# Strategic Combination of Theory, Plain Language, and Trusted Messengers Contribute to COVID-19 Vaccine Uptake: Lessons Learned from Development and Dissemination of a Community Toolkit

**DOI:** 10.3390/vaccines11061064

**Published:** 2023-06-05

**Authors:** Alison Caballero, Katherine J. Leath, Allie D. Staton

**Affiliations:** 1 Center for Health Literacy, University of Arkansas for Medical Sciences, Little Rock, AR 72212, USA; kjleath@uams.edu; 2 Arkansas Immunization Action Coalition, Little Rock, AR 72203, USA; allie@immunizear.org

**Keywords:** plain language, vaccine hesitancy, community health workers, community input, COVID-19, personal health literacy, community leaders, trusted messengers

## Abstract

Widely accepted practices for the development of health education materials include the use of theoretically driven content, the execution of plain language writing and design strategies, the solicitation of community input, and a plan for dissemination via trusted messengers. Here, we describe the development of a COVID-19 vaccine education toolkit and share preliminary outcomes from dissemination via community health workers. The toolkit was developed to equip community messengers to educate community members about the COVID-19 vaccine. It includes an easy-to-read workbook for community learners, a Leader Guide with scripting, and additional resources for community health workers and other local messengers. The Health Belief Model was used to select content for the workbook, which was refined with input from community members. A team of trained plain language writers worked with clinicians and subject matter experts to draft content that was deemed readable, understandable, and actionable by formal measures and drafts were further refined with additional community feedback. Survey results from community health workers who used the toolkit to provide local education about COVID-19 vaccines indicate that the toolkit facilitated confidence in their ability to deliver scientific content to their community members. More than two-thirds report that use of the toolkit facilitated community members’ decisions to receive COVID-19 vaccines.

## 1. Introduction

Personal health literacy, or “the degree to which individuals have the ability to find, understand, and use information and services to inform health-related decisions and actions for themselves and others” [1], is less than proficient for nearly nine in ten adults in the United States [2]. This results in challenges for individuals to find accurate health information, understand the content, and use it to improve or sustain personal health—or help others do so. These challenges are more profound in certain racial and ethnic minority groups and among those who are older or have limited income or education [2].

When the work described here was launched in December 2020, the impact of COVID-19 was growing exponentially. Between 19 September 2020 and 19 December 2020, new hospitalizations per week had increased nearly fivefold, from 22,314 to 102,168 [3]. The number of weekly deaths had similarly spiked over the same timeframe, from 4273 to 22,982 [4]. Like many other health conditions, COVID-19 has had a disparate impact on some populations. In 2020, reports showed increased risk for both hospitalization and death among older adults [5] and minorities, including African Americans [6]. Because these groups can both be challenged by limited health literacy [2] and are at increased risk for severe COVID-19 disease and poor outcomes, clear communication regarding strategies that individuals could employ to reduce their susceptibility to, and severity of, the disease was critical.

A key mitigation strategy for lessening the burden of the COVID-19 disease is widespread vaccination among the public. One month following the first vaccines being authorized for use in the United States, only about 1 percent of the adult population had completed an initial COVID-19 vaccine series [7]. This fell well short of the 70 to 85 percent necessary to reach protection through community immunity [8]. Further, many U.S. adults lacked the intention to get vaccinated. As intention is a consistent predictor of behavior uptake, this observation was particularly concerning. Among older adults and African Americans in Arkansas, who are more likely to suffer from both health literacy and undesirable outcomes from COVID-19, vaccination intention was low. In December 2020, many older Arkansans reported plans to never be vaccinated (13.4 percent) or responded as undecided (20.4 percent) [9]. The same survey suggested that among African Americans in the state of Arkansas, intention was far less, with 17 percent reporting they would never be vaccinated and 40.8 percent undecided [9]. 

Many theories attempt to predict or explain health behaviors. Theories include many components, or constructs, that together help health care and public health providers understand and shape individual health behaviors. One such theory is the Health Belief Model, which was introduced in the 1950s and has been widely used since [10]. The Health Belief Model focuses on individual health decision making and posits that intentions are influenced by beliefs about disease risk (susceptibility and severity), benefits and barriers to the recommended health behavior, prompts or cues to act, and self-efficacy that allows an individual to successfully engage in the behavior [10]. 

Despite the dire need to effectively educate older and minority populations about COVID-19 disease and risk mitigation strategies, many public health education efforts fell short. One study conducted early in the pandemic assessed a sample of online consumer education materials that were produced largely by public health entities. Authors found that most of the materials did not use plain language best practices to make them understandable and actionable and only 7 percent of materials in the study sample were assessed as “easy to read” [11]. Findings from this study were also observed in many similar studies of public- and patient-facing COVID-19 education [12,13,14]. These known challenges regarding vaccine uptake along with shortcomings in the health literacy responsiveness to vital pandemic communications informed and inspired the work described here.

In addition to the need for the use of plain language in the development of public health messages, the messenger who delivers the public health information is also an important consideration, especially in minority communities [15], where trust in health information may be suboptimal. As such, a growing body of literature supports the use of trusted local messengers to deliver public health education [16,17]. One example of trusted local messengers are community health workers (CHWs), defined as “trusted members of local communities who share lived experiences with their neighbors and peers, and they are experts in navigating complex systems of care, serving as a link between clinical and community-based services and the people who need them most” [18]. The literature suggests that health information delivered by those considered “outsiders” to a community, including public health officials, may not be as successful in sharing health information compared to instances when the information is delivered by those “inside” the community. As “insiders”, CHWs may be more optimally equipped to serve as messengers for delivering public health information. CHWs were engaged during the pandemic to assist with public health education by helping create and disseminate health education materials that are culturally relevant [19].

Given the critical nature of pandemic-related public health communications for older and minority groups at elevated risk for severe COVID-19 illness and limited health literacy, a collaborative team formed to develop and disseminate COVID-19 vaccine information in communities across Arkansas. Here, we summarize outcomes from this effort and provide an in-depth description of the evidence-based processes used, including the use of survey data and health behavior theory to select content; partnerships with subject matter experts and community members to shape content; the contributions of a plain language writing team to optimize the degree to which educational content was readable, understandable, and actionable; and community dissemination, engaging community health workers as local trusted messengers. This approach can be replicated and applied by public health and health care institutions as they develop and deliver health education content on a variety of health-related topics to improve health and advance health equity.

## 2. Materials and Methods

### 2.1. About the Toolkit

The purpose of this project was to equip community leaders with a toolkit [20] to educate community members on the safety and effectiveness of COVID-19 vaccines and help inform individual decision making around vaccine uptake. The toolkit was designed between December 2020 and February 2021 for trusted messengers to deliver information about COVID-19 vaccines in their own communities. Staff at a health literacy center at an academic medical center were approached and engaged by leaders of a state department of health to develop the toolkit in late 2020, before the first COVID-19 vaccines were authorized for use. The early timing of this outreach by the state health department was due to the anticipation of the widespread outreach needed to promote vaccine uptake.

The original toolkit included a participant workbook (COVID-19 Vaccine: Learn the Facts to Stay Safe and Protect Others) along with companion materials, which included a leader guide and leader script. The participant workbook is the foundation of the toolkit and was designed to be interactive. It includes checkboxes and fillable blanks to provide learners with an opportunity to internalize the content and to promote individual decision making. As an example, in the section describing factors that increase risk for severe COVID-19 (e.g., smoking or being African American), the list includes checkboxes, and learners are encouraged to check the risks that apply to them. The leader guide and script are additional resources for trusted messengers to use when presenting the workbook content in a community group learning session. 

### 2.2. Workbook Content Selection Using Health Behavior Theory

To develop the workbook, plain language experts at the health literacy center drafted key messages and created a content outline which addressed relevant constructs of the Health Belief Model [10]. Key messages for each construct were aligned with vaccine intent surveillance data [9]. See Table 1 for a list of constructs and associated key messages.

### 2.3. Workbook Content Development 

After subject matter experts approved the outline, a small community panel was recruited and convened to provide formative feedback. The panel included African Americans and older adults to ensure that the team received input on the perceived severity of and susceptibility to COVID-19 and barriers and the benefits of getting a COVID-19 vaccine from these specific populations. Feedback from the input session was incorporated into the workbook outline. 

Once the outline was finalized, plain language experts drafted content and planned for document design using plain language best practices [21]. Examples of plain language best practices that guided this work include the use of short sentences and words, avoiding scientific and health industry jargon, expressing numbers as whole numbers rather than percentages, and making learners’ suggested action steps clear and detailed. Drafted content was formally assessed by the writing team for readability, understandability, and actionability. 

### 2.4. Workbook Content Plain Language Assessment

Beginning with the initial draft, iterations of the workbook were formally assessed by the writing team for readability to ensure the workbook was considered easy to read. Using standardized processes, staff selected and cleaned samples of text. For materials over eighteen hundred words, staff selected at least three samples of six hundred words each from the beginning, middle, and end of each material, ensuring that the text they selected was representative of the material and did not include information that was overly simple or difficult. For shorter materials, the entire body of text was used in the assessment. To clean the samples, staff removed bullets and numbers at the start of lists, removed punctuation that did not denote the end of a sentence, and added punctuation at the end of titles, headers, and bullets. This cleaning process ensured that the readability software correctly identified the end of each sentence. Staff inserted cleaned content samples into Seven Formulas software (Micro Power & Light Co., Dallas, TX, USA) to generate results from three validated formulas, namely Flesch-Kincaid [22], SMOG [23], and Fry Graph [24], then averaged the three scores to arrive at a mean readability score. The mean was then used to categorize the material into levels of difficulty: easy (under seventh grade), average difficulty (seventh through ninth grade), or difficult (tenth grade and above). 

Because readability alone does not ensure comprehension, the health literacy team further assessed the iterations of the workbook content for understandability and actionability. Trained staff used the Patient Education Materials Assessment Tool for Print Materials for Print (PEMAT-P) [25] to assess these domains of plain language attributes. The PEMAT-P includes seventeen items to assess understandability and seven items to assess actionability and produces separate percentage scores for each area. Health literacy staff used a standardized procedure, including independent reviews by two reviewers who rated materials in accordance with the instrument’s user guide. Reviewers then met to discuss their rating differences and to come to a consensus. Findings from the PEMAT-P assessment were used to make improvements to the workbook before field testing with community members.

### 2.5. Workbook Field Testing

**Figure 1:**
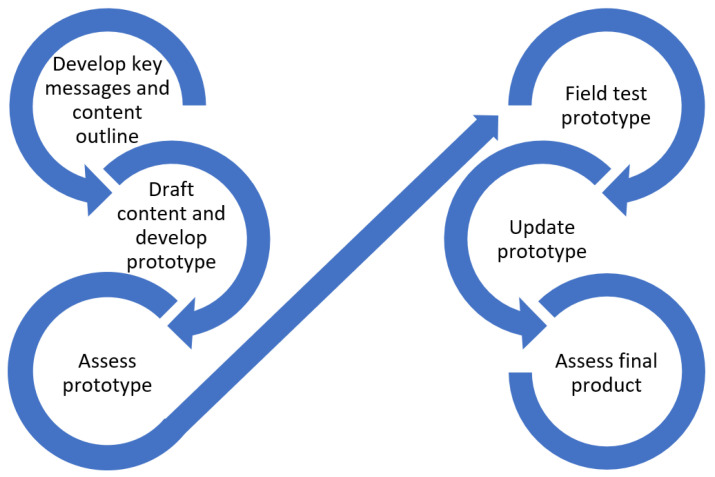
Iterative Materials Design Process.

After workbook content was approved by subject matter experts, as a part of the health literacy center’s iterative design process (See Figure 1), the development team conducted a virtual field testing session to capture potential users’ perspectives about the workbook’s organization, design, formatting, tone, and ease of comprehension. Staff recruited participants from an existing database of community members who previously agreed to take part in field testing sessions. To ensure the inclusion of individuals who are at risk for limited health literacy, we screened participants using the validated Newest Vital Sign tool [26]. Before the session, participants were sent the workbook material to review and given instructions to identify items and concepts that authors made hard to understand or that could otherwise be improved. 

The session was conducted via Zoom and included a total of seven participants. All were female, four were African American, one was Native American, and one was multi-racial. A trained facilitator conducted the session using a prepared facilitator’s guide. The discussion focused on the items participants identified during their independent review of the material, with a focus on how each item should be improved. As participants voiced that a certain word, phrase, or concept needed improvement, discussion focused on their preferences for those improvements. 

A summary report and recommendations for updates to the workbook were presented to the subject matter experts whose approved edits were incorporated into the final workbook. One example of the recommendations from field testing was to include a recommendation for readers to ask their doctors about ingredients in the vaccine. Ingredients were not listed in the original draft, and one participant suggested we add it. Realizing that the list would vary by manufacturer, that the list would be long and thus make the overall piece longer, and that participants would not recognize many ingredient names, the session leader encouraged participants to discuss other ways to address the perceived barrier that the vaccine contained unknown and potentially unsafe ingredients. 

### 2.6. Toolkit Supplementary Materials Development

After the community workbook was finalized, a complementary leader guide, script, and slide deck were developed using plain language best practices. The original goal of these companion materials was to help trusted leaders facilitate group learning sessions with community members. The original toolkit (which included the workbook leader guide with script and slide deck) was published in February 2021. In addition to paper copies of the workbook being printed and distributed throughout the state by the state’s department of health, the toolkit was posted on a publicly accessible state immunization coalition website. 

As information about the COVID-19 vaccine changed, the workbook was updated, and a data sheet was added to the toolkit to eliminate the need for updates to the workbook based on changes to current data. The data sheet accompanies the workbook and is updated regularly with the most current COVID-19 statistics, including hospitalization and death rates by race and age. The updated workbook currently available contains “blanks” for participants to add current statistics from the updated data sheet as these are shared by the community messenger. Because the users of the toolkit were not using the slide deck, it was not updated or included in future editions of the toolkit. 

### 2.7. Toolkit Dissemination

In spring of 2022, the state immunization action coalition collaborated with a group of approximately 150 community health workers (CHWs) who were tasked with improving COVID-19 vaccine education efforts throughout the state. This collaboration was initiated by a pharmacist serving as the Vaccine Confidence Strategist for the state immunization action coalition, who had had many unsuccessful attempts at hosting community workshops with the COVID-19 toolkit. It became apparent that a local trusted messenger was needed to deliver these messages. Additionally, while the original intent was for the toolkit’s content to be delivered in group community learning sessions, the pandemic posed challenges to group gatherings.

The pharmacist hosted training sessions with these 150 CHWs on toolkit utilization to increase their confidence in discussing COVID-19 vaccines with their community members. Training sessions included the in-depth discussion of each part of the toolkit (workbook, data sheet, leader guide, and script), with emphasis on the workbook content and addressing frequently asked questions. CHWs were encouraged to utilize the workbook to facilitate either group or individual discussions with community members. They were discouraged from using the workbook simply as a “handout” without discussion.

CHWs were encouraged during the initial training sessions and follow-up emails to submit an online survey each time they used the workbook. Completing the survey was not a requirement for using the workbook; thus, data collection was limited to those who opted in. The survey was designed to assess workbook utilization and determine perceptions regarding its value and outcomes among CHWs. See Table 2 for a complete list of questions and answer choices from the survey.

## 3. Results

### 3.1. Toolkit Materials Assessment

The results of the readability and PEMAT-P plain language assessments of toolkit materials are provided in Table 3. The grade level range across all materials was fourth to ninth grade, with mean readability calculated as grade seven. The community-facing workbook registered as “easy to read” and leader materials registered at an average level of difficulty. High percentage scores from the PEMAT-P in both the understandability and actionability domains indicate that the materials were written in a way that is easy for a lay audience to understand and act on. Toolkit documents earned a score of 100 percent for understandability and actionability, with one exception: reviewers noted in the PEMAT-P assessment that using icon arrays in the data sheet “Learn the Risk of Getting Sick with COVID-19” would make the numbers more understandable. Because icon arrays would need to be updated along with data each time new data was available, and the expectation was that this would occur frequently, the team opted against implementing this suggestion so the data sheet could be updated easily.

### 3.2. Community Health Worker Survey Results

**Figure 2:**
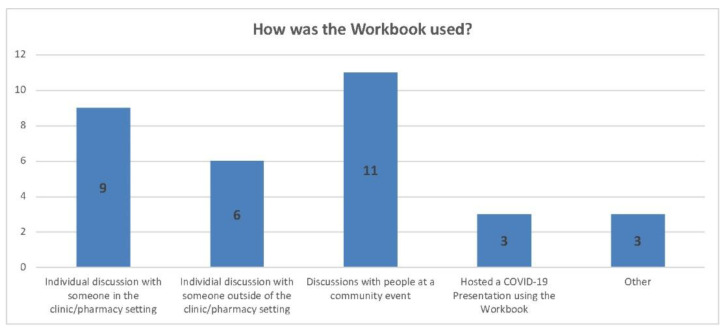
Workbook Use.

A total of thirty-two individual uses of the workbook were reported via twenty-four responses. While the survey was available online and could be completed by any user of the toolkit, CHWs who attended training were encouraged to respond and thus nineteen of the twenty-four responses were from CHWs. Most CHWs used the workbook to discuss COVID-19 vaccines with individuals, rather than in a group presentation. Three respondents reported in their comments that they did not physically use the workbooks (by using them as handouts), but the information they learned from the workbook helped them to better understand COVID-19 vaccines in a way that helped them explain it to others (see Figure 2). All but one respondent reported feeling more confident in discussing COVID-19 vaccines when using the workbook.

**Figure 3:**
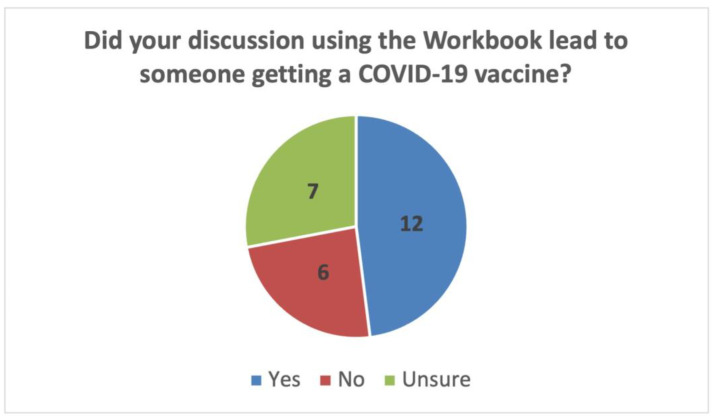
Perceptions of Workbook Outcome.

Though it cannot be determined that the information from the workbook is solely responsible for individuals choosing to receive a COVID-19 vaccine, the majority of respondents reported that community members chose to receive a COVID-19 vaccine after speaking with someone who utilized the workbook. Only five respondents felt that their discussion did not lead to learners opting for vaccination (See Figure 3).

Results for each survey item are included in Table 4 and Table 5 below.

## 4. Discussion

Controlling the spread of communicable disease within a community requires individual contributions to preventive measures. As COVID-19 numbers grew, several mitigation strategies were suggested to individuals (e.g., social distancing, mask wearing, and proper hand hygiene), but vaccination remained a critical element for containment. While individual choice surrounding health behaviors is complex, as indicated by a host of health behavior theories with multiple constructs that interact with one another, information sharing is at the core of many of those constructs. As an example, theoretical constructs often point us toward shifting attitudes (i.e., about the severity of disease or the value of promoted health behaviors), but attitudes are influenced heavily by the clarity of information received about those topics and trust in those delivering the message. As vaccines were being developed, tested, and authorized for use, information on these topics was plentiful if not overwhelming. Despite mixed evidence as to the power of individual constructs within the Health Belief Model to impact intention and behavior [27], we centered our messaging on the constructs’ additive benefits. A systematic review of over one hundred studies focused on the use of the Health Belief Model in COVID-19 vaccine uptake programs found that constructs’ impacts varied by specific population and differed when examining intention to attain the primary vaccine series as opposed to a booster dose [28]. Still, the review concluded that the Health Belief Model remains a useful tool for predicting vaccine intention.

The information was new to community members and often (as previously described) not written in ways that promote understanding or action. In addition, consumers were burdened with conflicting information and the task of discerning which information was credible. Our approach of combining plain language content vetted by community members with the delivery of messaging led by CHWs, as trusted messengers can help overcome those challenges.

Our work is not without limitations. Data presented were collected from a small number of respondents, primarily CHWs, who were extended a personal invitation by the immunization action coalition team to complete the optional online survey and thus may not be generalizable to other populations of messengers who downloaded or used toolkit materials. Additionally, the survey did not ask respondents to report demographic information about themselves or the demographics of the people they spoke with, so we cannot make conclusions about how the toolkit’s material supported decision making among specific groups with known health literacy challenges or evidence of enhanced vaccine hesitancy or about how representative respondents are of the populations they serve.

Another limitation is that responses are self-reported and actual vaccinations cannot be verified at the individual level. However, CHWs were required to report the precise number of immunizations given at each community event in which they participated, which leads us to believe that the data reported in the survey are accurate.

A final limitation is that we cannot attribute all the instances of the successful vaccine uptake reported to the teaching conducted by CHWs in general or to CHWs’ use of toolkit materials specifically. As behavior change is complex, theoretical constructs not addressed by the toolkit could have been more impactful drivers of vaccine uptake.

In consideration of future impact, the results observed in this project may not be replicable in other public health efforts. The project described here was focused tightly on community education on the singular public health topic of COVID-19. When considering the opportunity to apply the approach described here to other public health education needs that are not as far-reaching or novel as COVID-19, it is possible that motivation to plan for, develop, and disseminate educational programming with such robust methods may be lacking. Because public health education is an ongoing necessity to support the prevention and management of a wide range of both chronic and infectious diseases, the exploration of the elements of the comprehensive approach described here could be a worthwhile investment.

## 5. Conclusions

Public health education is an essential component of managing the spread of both infectious and chronic diseases and is often designed with a goal of impacting individual health behaviors. While individual decision making regarding preventive behaviors is complex, the combination of content guided by health behavior theory and tailored through community input, expert plain language writing, and delivery by a trusted messenger can be powerful. Our work demonstrated that the educational content produced and delivered in this fashion supported desirable decision making regarding vaccination against COVID-19.

The need for this will work will be ongoing, as only 69.5 percent of U.S. adults have received a primary COVID-19 vaccination series as of May 2023 [29]. As research unveils additional information about long-term impacts of COVID-19, new public health education will be required. Information development and dissemination strategies such as those described here can help all health professionals, including CHWs, keep the public updated on the most current and accurate information. Beyond COVID-19, this combination of strategies can be employed to support health education campaigns on many other topics.

## Figures and Tables

**Table 1:** Workbook Key Messages and Alignment with Health Belief Model Constructs.

Constructs of the Health Belief Model	Key Messages
Perceived susceptibility (Perception of disease risk)	Older and minority groups are at an increased at risk of getting a COVID-19 infection.
Perceived severity (Perception of the severity of the disease)	COVID-19 infections can cause a range of symptoms, sometimes leading to hospitalization, death, or permanent health problems. Older adults and minorities are at an increased risk of these severe outcomes. No individual can predict how severely a COVID-19 infection will affect them.
Perceived barriers (Perception of barriers to getting the vaccine)	The methods for developing and authorizing COVID-19 vaccines were expedited but no steps were skipped in the process. Testing for safety was carried out and included older adults and minorities. COVID-19 vaccines are free for everyone in the United States, regardless of insurance status or U.S. citizenship
Perceived benefits (Perception of benefits of getting a vaccine)	Getting a COVID-19 vaccine can protect not only the individual receiving the vaccine but also family and friends. This includes protecting those who are unable to get a COVID-19 vaccine. Getting a COVID-19 vaccine can limit the need to miss work or school, thus supporting financial and academic goals.

**Table 2:** COVID-19 Vaccine Workbook Utilization Survey.

Survey Question	Survey Response Options
What is your practice location type?	⚬ Clinic ⚬ Pharmacy ⚬ AFMC ⚬ Other (please specify)
Your Organization/Clinic/Pharmacy Information	⚬ Your Name (First and Last) ⚬ Name of Organization/Clinic/Pharmacy ⚬ Address of Organization/Clinic/Pharmacy ⚬ City/Town ⚬ State/Province ⚬ ZIP/Postal Code ⚬ Your Email Address
Name of person who used the Workbook (First and Last Name)	(Free Text)
Date Workbook was used	(Free Text)
Title of person who used the Workbook	⚬ Community Health Worker ⚬ Nurse ⚬ Physician ⚬ Pharmacist ⚬ Other (please specify)
Which language version of the Workbook was used?	⚬ English ⚬ Spanish ⚬ Marshallese
How was the Workbook used? (please complete a separate survey for different uses of the Workbook)	⚬ Individual discussion with someone in the clinic/pharmacy setting ⚬ Individual discussion with someone outside of the clinic/pharmacy setting ⚬ Discussions with people at a community event ⚬ Hosted a COVID-19 presentation using the Workbook ⚬ Other (please specify)
How many people did you speak with during this event? (an event can be an individual conversation with someone)	⚬ 1 ⚬ 2–4 ⚬ 5–9 ⚬ 10–19 ⚬ 20 or more
Did using the Workbook help you feel more confident in talking to people about COVID-19 and COVID-19 vaccines?	⚬ Yes ⚬ No ⚬ If no, please explain.
Did your discussion using the Workbook lead to someone getting a COVID-19 vaccine?	⚬ Yes ⚬ No ⚬ Unsure
How many people received a dose of a COVID-19 vaccine after you spoke with them? (please type “0” if no people received a dose after you spoke with them)	(Free text)
Were you asked any questions during the event that you felt unprepared to answer? If yes, please specify.	⚬ No ⚬ Yes (please specify)
Do you have any suggestions for improving the COVID-19 Workbook/Toolkit? Please answer honestly if you have a suggestion. We welcome constructive criticism.	(Free text)
Success Stories: If you have a specific success story you would like us to know about, please feel free to share it with us.	(Free text)
Learning Experience: If you have a specific experience that you learned from and would like us to know about, please feel free to share it with us.	(Free text)
Other Comments: If you have other comments for our team, please feel free to share them below.	(Free text)

**Table 3:** Results from Formal Plain Language Assessments of Toolkit Content.

Document Title	Grade Level Readability Range	Level of Difficulty	Pemat-P Understandability Score	Pemat-P Actionability Score
COVID-19 Vaccine: Learn the Facts to Stay Safe and Protect Others—Workbook	4th to 7th	Easy to read	100%	100%
COVID-19 Vaccine: Learn the Facts to Stay Safe and Protect Others—Leader Guide	6th to 8th	Average difficulty	100%	100%
COVID-19 Vaccine: Learn the Facts to Stay Safe and Protect Others—Leader Script	5th to 8th	Average difficulty	100%	100%
Learn the Risk of Getting Sick with COVID-19–Data Sheet	5th to 9th	Average difficulty	92.3%	100%

**Table 4:** Responses to COVID-19 Vaccine Workbook Utilization Survey.

Survey Question	Responses (n = 24)		
	Response Options	Frequency	Percentage
What is your practice location type?	Clinic	0	0%
	Pharmacy	12	50.0%
	AFMC	12	50.0%
	Other	0	0%
Title of person who used the Workbook	CHW	19	79.2%
	Nurse	0	0%
	Physician	0	0%
	Pharmacist	3	12.5%
	Other	2	8.3%
Which language version of the Workbook was used?	English	23	95.8%
	Spanish	0	0%
	Marshallese	1	4.2%
How was the Workbook used? (please complete a separate survey for different uses of the Workbook) *	Individual discussion with someone in the clinic/pharmacy setting	12	50%
	Individual discussion with someone outside of the clinic/pharmacy setting	3	12.5%
	Discussions with people at a community event	10	41.7%
	Hosted a COVID-19 Presentation using the Workbook	3	12.5%
	Other	3	12.5%
How many people did you speak with during this event (an event can be an individual conversation with someone)?	1	6	25%
	2 to 5	7	29.2%
	6 to 9	1	4.2%
	10 to 19	5	20.8%
	20 or more	5	20.8%
Did using the Workbook help you feel more confident in talking to people about COVID-19 and COVID-19 vaccines?	Yes	22	91.7%
	No (please explain) **	2	8.3%
Did your discussion using the Workbook lead to someone getting a COVID-19 vaccine?	Yes	12	50.0%
	No	5	20.8%
	Unsure	7	29.2%
Were you asked any questions during the event that you felt unprepared to answer? If yes, please specify.	Yes (please explain)	1 (Not always able to answer extreme conspiracy theory driven questions)	4.2%
	No	23	95.8%

* Some respondents selected more than one response. ** No respondents entered explanatory text.

**Table 5:** Free Text Responses to COVID-19 Vaccine Workbook Utilization Survey.

Survey Question	Responses (Frequency)	Representative Quote(s)
How many people received a dose of a COVID-19 vaccine after you spoke with them? (please type “0” if no people received a dose after you spoke with them)	0 (10) 1 (3) 3 (1) 4 (1) 5 (1) 15 (1) 16 (1) 18 (1) 25 (1) Unsure/unknown (4)	N/A
Do you have any suggestions for improving the COVID-19 Workbook/Toolkit? Please answer honestly if you have a suggestion. We welcome constructive criticism.	N/A	“I like the workbook. I never had the opportunity to present a class but practiced a lot with it. I think people would be able to easily follow the presentation”.
Success Stories: If you have a specific success story you would like us to know about, please feel free to share it with us.	N/A	“Once I have had the discussion of the importance of the vaccine, I have never had anyone turn me down”.
Learning Experience: If you have a specific experience that you learned from and would like us to know about, please feel free to share it with us.	N/A	“I learned a lot about the workbook and the motivational aspect on how to be comfortable talking to the community”.
Other Comments: If you have other comments for our team, please feel free to share them below.	N/A	“I think the workbook is a great tool. Very easy to use, read and understand. Great information in it”. “Valuable tool and very useful for seminars and educational gatherings”. “I learned a lot with the vaccine webinars. It made me comfortable and happy with talking to the community”.

## Data Availability

Raw data from the readability and PEMAT-P assessments of toolkit materials are available by email request (KJLeath@uams.edu). Raw survey data are also available by email request (allie@immunizear.org).
